# Morphology of the normative human cone photoreceptor mosaic and a publicly available adaptive optics montage repository

**DOI:** 10.1038/s41598-024-74274-y

**Published:** 2024-10-05

**Authors:** Robert F. Cooper, Snega Kalaparambath, Geoffrey K. Aguirre, Jessica I. W. Morgan

**Affiliations:** 1 Joint Department of Biomedical Engineering, Marquette University and Medical College of Wisconsin, Milwaukee, USA; 2 Department of Ophthalmology, Medical College of Wisconsin, Milwaukee, USA; 3 Scheie Eye Institute, Department of Ophthalmology, University of Pennsylvania, Philadelphia, USA; 4 Department of Neurology, University of Pennsylvania, Philadelphia, USA; 5 Center for Advanced Retinal and Ocular Therapeutics, University of Pennsylvania, Philadelphia, USA

**Keywords:** Retina, Imaging and sensing

## Abstract

Adaptive optics ophthalmoscopy has enabled visualization of the in vivo human photoreceptor mosaic in health, disease and its treatment. Despite this, the clinical utility of the imaging technology has been limited by a lack of automated analysis techniques capable of accurately quantifying photoreceptor structure and a lack of an available normative image database. Here, we present a fully automated algorithm for estimating cone spacing and density over a complete adaptive optics montage along with a database of normative images and cone densities. We imaged the cone mosaics surrounding the fovea and along the horizontal and vertical meridians of fifty normal-sighted controls with a custom-built, multimodal adaptive optics scanning light ophthalmoscope. Cone spacing was automatically measured in the frequency domain and spacing measurements were converted to estimates of cone density at all locations across the montage. Consistent with previous reports, cone density measurements were highest near fovea (152,906 ± 53,209 cones/mm^2^) and decreased exponentially with eccentricity. A 2.5-fold variation was found in cone density estimates at 0.1 mm, this variation decreased to 1.75-fold at 1 mm. We provide all images, mosaic quantifications, and automated software open source. This database will aid investigators in translating adaptive optics ophthalmoscopy to clinical applications.

**Supplementary Information:**

The online version contains supplementary material available at 10.1038/s41598-024-74274-y.

## Introduction

Adaptive optics (AO) ophthalmoscopy has enabled noninvasive observation of subcellular retinal structure for more than a quarter century^1^. The technique measures and compensates for the optical aberrations present in the living eye; this is often (but not always) achieved through use of a wavefront sensor for measuring aberrations, and a deformable mirror for correcting aberrations. By incorporating aberration correction in ophthalmic imaging systems such as scanning light ophthalmoscopes, noninvasive retinal imaging has approached the resolution expected for diffraction limited imaging through the dilated pupil of the living eye. Diffraction-limited imaging enables investigators to observe the structure and function of individual cells within the retina in both cross-sectional and longitudinal studies, and to study cellular integrity in health, disease, and treatment.

Despite the capabilities of AO-enhanced imaging, the clinical utility and thus wide-spread adoption of the technique has been hindered in part by the lack of automated tools available for quantification of features visible in AO montages; in particular, the photoreceptor mosaic. For example, current state of the art methods for assessing cone mosaic structure in AO ophthalmoscope images involves extraction of small regions of interest (ROIs) followed by semi-automated cone identification algorithms for identifying cell locations, followed by analysis of the mosaic in each ROI^2,3^. The entire process is inherently sensitive to selection bias, meaning that all mosaic quantifications and interpretations are bound to the operator’s choice of ROI and cone locations. The vast majority of studies circumvent this by ensuring that all data is selected by a single individual, or a group of individuals that are then assessed using an inter-grader agreement measure such as an interclass correlation coefficient^2,4–7^.

Hidden in results of an AO ophthalmoscopy study is the significant amount of training and labor necessary to complete the processing and analysis of the image data. Training individuals to be consistent and accurate ROI and cone identifiers is an extensive process that requires years of experience and training^4^. Further, despite advances in photoreceptor identification, the sensitivity and specificity of algorithms available to date have not enabled fully-automated analysis pipelines. The clinical utility of AO ophthalmoscopy is further hampered by the lack of substantive, publicly available, normative datasets, inclusive of AO images. This represents a significant limitation for effective comparisons across research groups and restricts analyses of abnormal retinal findings. Moreover, it hampers the development of AO-centric algorithms by groups that do not possess an AO ophthalmoscope. Thus, there is a substantial need for algorithms capable of quantifying the photoreceptor mosaic structure without operator input, and also for a publicly available normative database of quantified photoreceptor mosaic images.

In this work, we present a fully automated algorithm for estimating local cone photoreceptor mosaic density over a complete, montaged AO dataset. We provide normative analyses for total cone counts and cone density versus retinal eccentricity for all four retinal meridians attained from 50 eyes of 50 participants with no known retinal or corneal pathology. In addition, we include an open-access database of the multi-modal AOSLO montaged images, and the automated analysis results from each montage. In addition to providing quantitative measures of normative photoreceptor mosaic morphology only enabled by a dataset of this scale, it is our hope that these data spur the development of novel algorithms and normative comparisons for the broader AO ophthalmoscopy community.

## Results

**Fig. 1:**
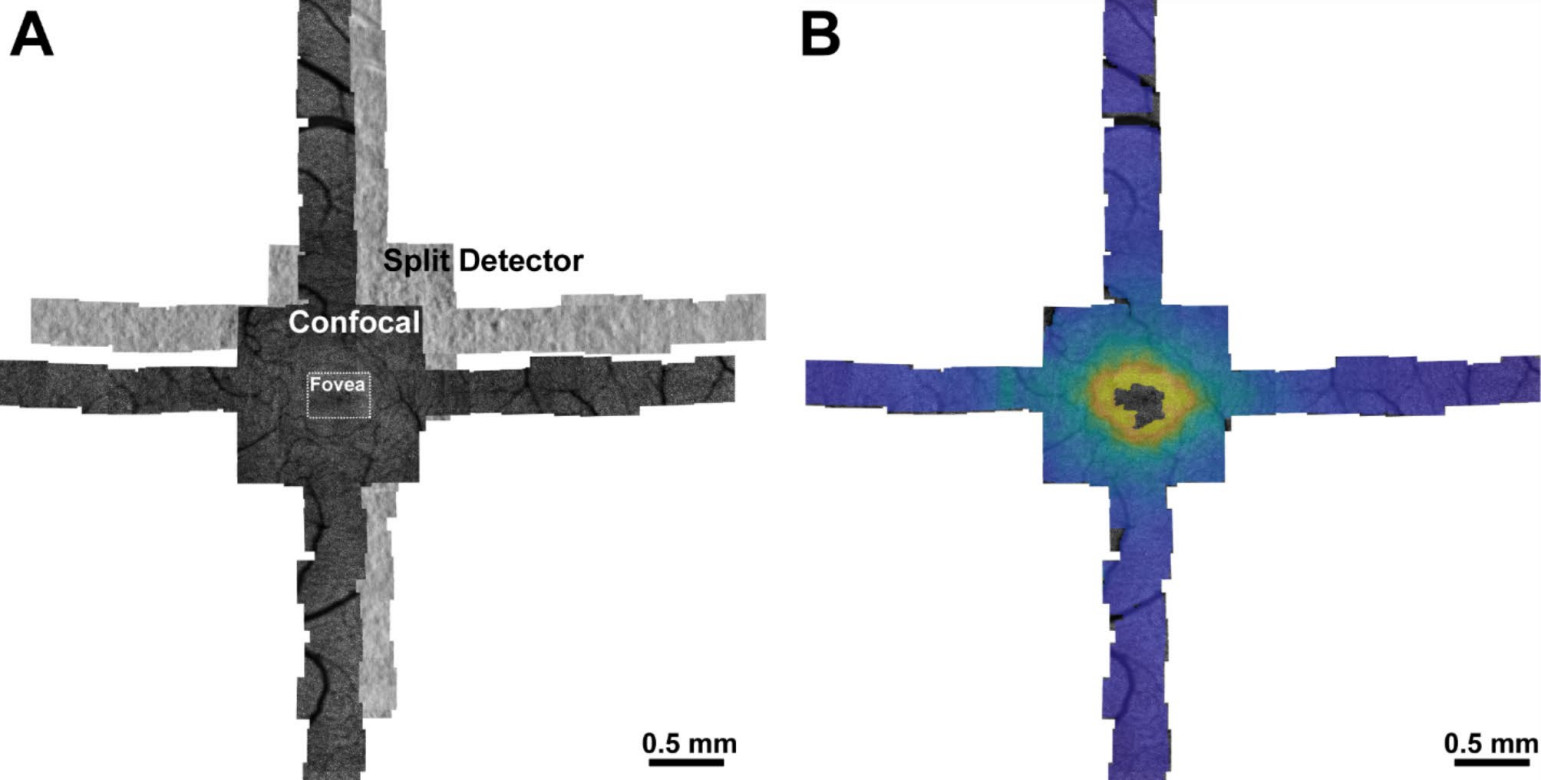
Example multi-modal AOSLO montage and cone density estimations from participant 11,002. Split detection and confocal AOSLO images depict the photoreceptor inner segment and waveguiding outer segment reflectance respectively (**A**). Cone spacing was measured and converted to an estimated cone density for each point in the montage (**B**). Higher cone densities correspond to warmer colors.

Fifty normal sighted control participants aged 18–67 years (mean 27.8 years, standard deviation 9.5 years) participated in the study (Supplemental Table 1). All participants had best corrected visual acuities of 20/25 or better. Participant axial lengths ranged from 22.11 to 27.52 mm (mean 24.34 mm, standard deviation 1.35 mm) and had a range of corrective prescriptions from − 11.25 D to + 0.75 D (Supplemental Table 1). A single, randomly selected eye from each participant was successfully imaged, registered, montaged and analyzed (Fig. 1). Foveal centers were automatically found in all but two participants (11065, 11081). For these participants, the algorithm automatically detected that it was unable to successfully find the foveal center, and therefore prompted the user (RFC) to draw a rectangular box encompassing the fovea and parafoveal region, from which the foveal center was then automatically determined using the same method as for the full montage. The cone densities for almost all individuals from confocal and split-detection modalities were successfully blended according to the average radial confidence of the montage (see “Methods” section). Eight participants had algorithm confidence that was similar between the confocal and split-detection modalities. In these cases, the blend radius was selected from the location with the highest similarity between the two modalities.

**Fig. 2:**
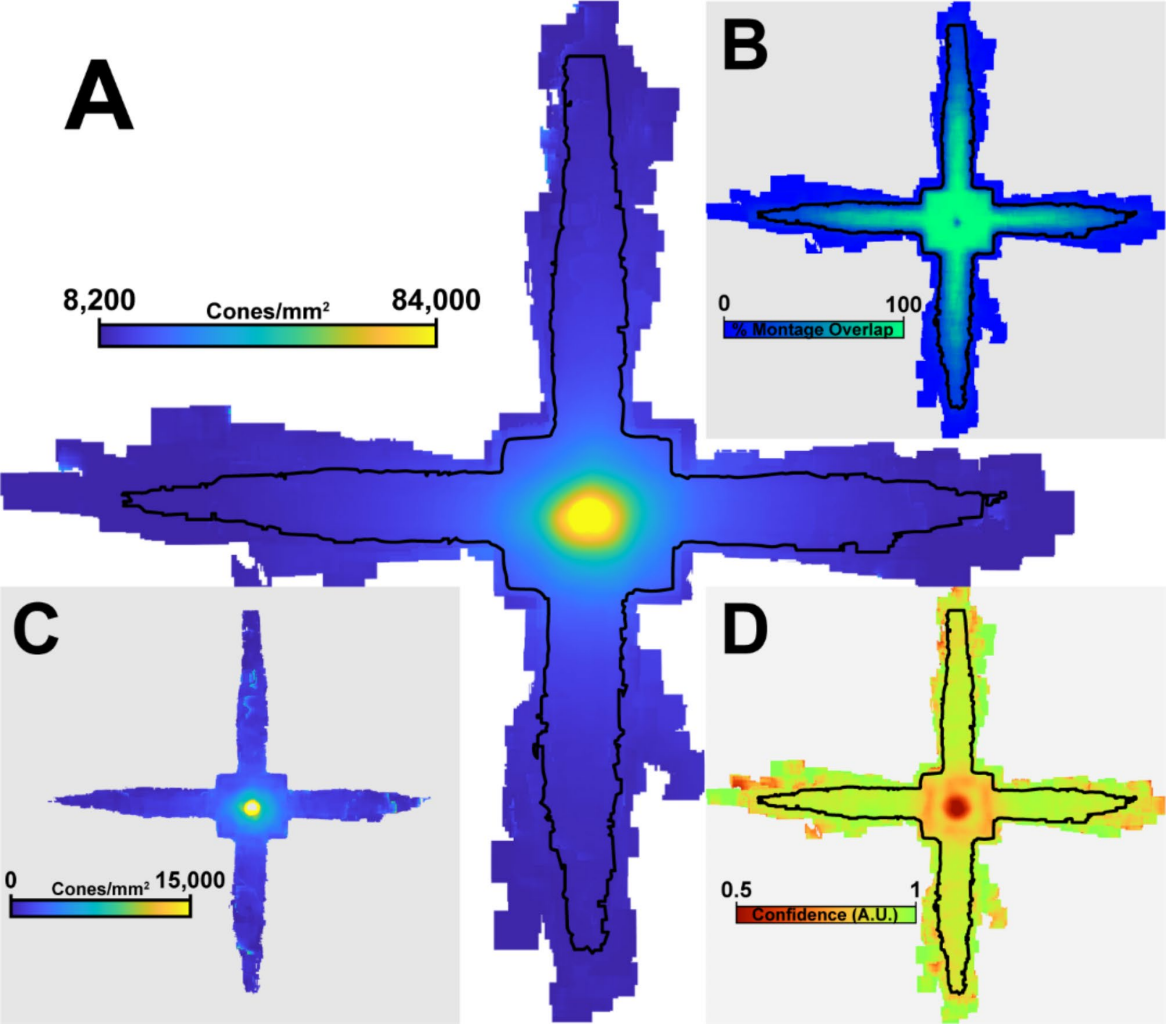
Average density (**A**), overlap map (**B**), density standard deviation (**C**) and confidence measure (**D**) compiled from all 50 AOSLO montages. The black outline corresponds to the region of the montage containing more than 10 participants.

Retinal coverage was largely consistent across participants, enabling us to summarize density, and algorithm confidence (Fig. 2). Moderate inter-participant variability in the rotational axis of each participants’ meridian strips lead to a reduced analyzable region (defined as > 10 participants) in retinal locations distant from the foveal center (Fig. 2B). This resulted in a 53.7% reduction in total coverage, though most excluded areas were data from single participants and did not preclude detailed analysis along each meridian. Across all montages, confidence was highest in the perifoveal macula from 1 to 2 mm eccentricity, with a decrease in confidence observed at the fovea, around montage edges, and at the most distant eccentricities (Fig. 2D).

**Fig. 3:**
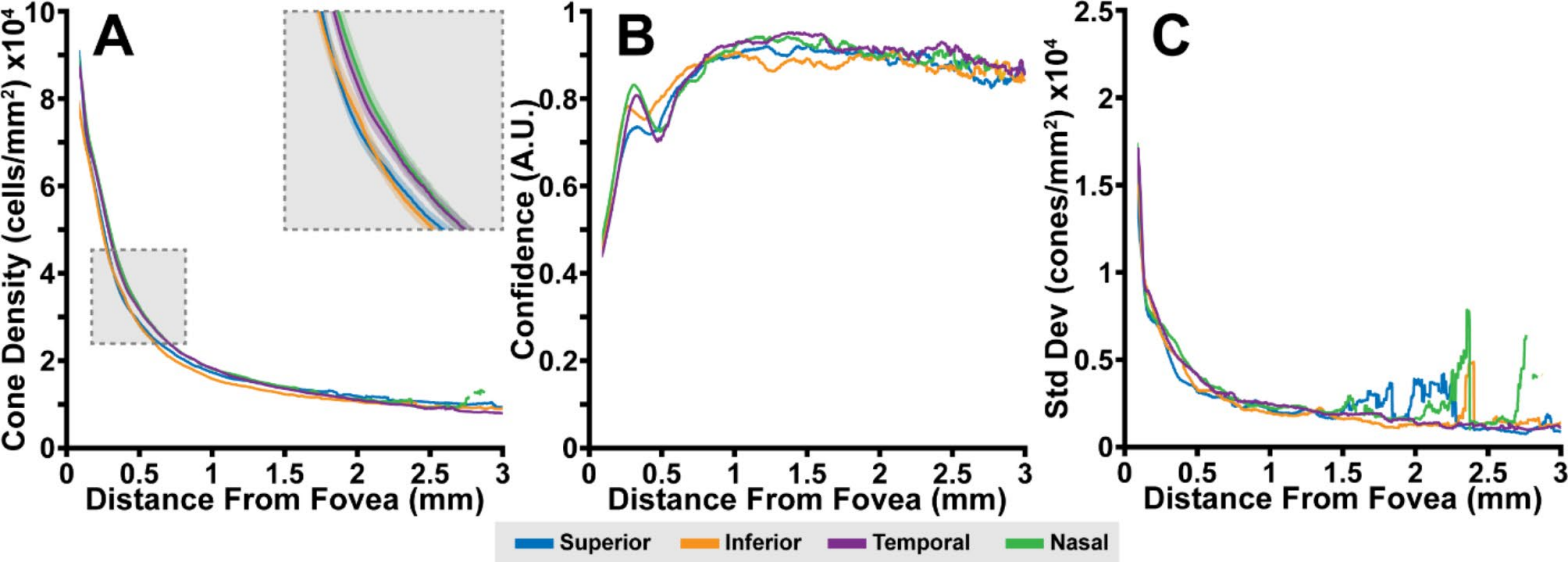
Average cone density (**A**), confidence (**B**), and standard deviation (**C**) from all 50 participants versus eccentricity from the fovea for all four cardinal meridians. Cone density measurements in this study agreed with previous reports and demonstrated a clear separation between the horizontal and vertical meridians (**A** inset; shaded regions are the 95% confidence interval for each meridian). Standard deviation was generally higher in the horizontal meridians near the center of fixation, becoming increasing noisy with eccentricity.

**Fig. 4:**
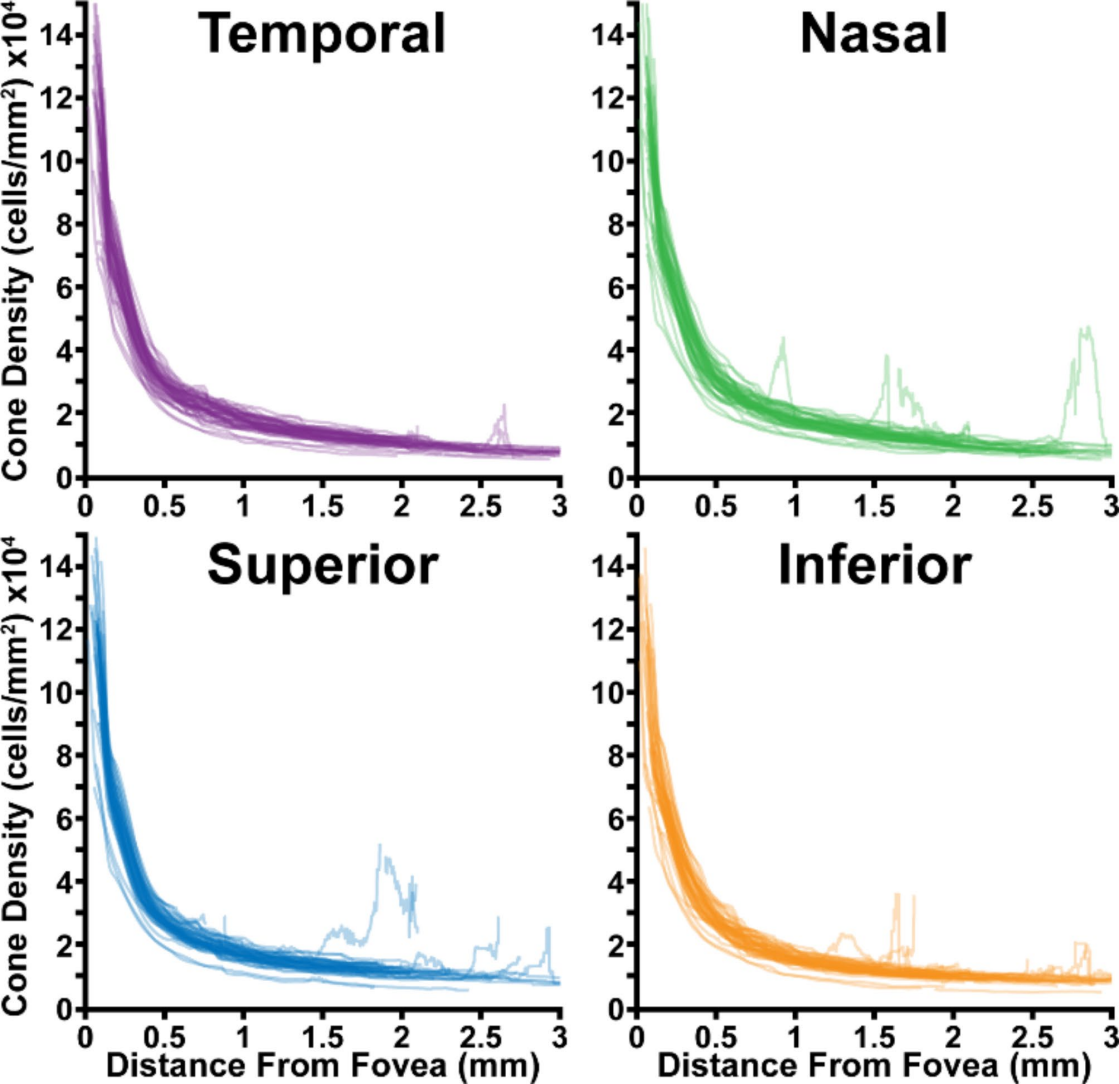
The individual density curves of all 50 participants as a function of eccentricity, grouped by meridian.

In each participant and in the 50 participant average, density along the meridians followed the previously reported relationship as a function of eccentricity (Figs. 3A and 4)^8–12^. Consistent with prior observations, greater inter-participant variability was observed closer to the fovea (standard deviation: >15,000 cones/mm^2^ at 0.1 mm) than at greater eccentricities (standard deviation: ~2,000 cones/mm^2^ at 3 mm; Fig. 4C). Further, average density was observed to be consistently higher along the horizontal (temporal and nasal) meridians in comparison to the vertical (superior and inferior) meridians (Fig. 3A), and this was roughly mirrored in density standard deviation (Fig. 3C). Conversely, meridian-averaged confidence increased logarithmically as a function of eccentricity, starting at ~ 0.6 A.U. near the fovea, and reaching ~ 0.9 A.U. at greater eccentricities (Fig. 3B). This is consistent with a priori observations of images obtained near the fovea, and images of densely packed foveal cone photoreceptors that are at the edge of this AOSLO’s resolution.

**Fig. 5:**
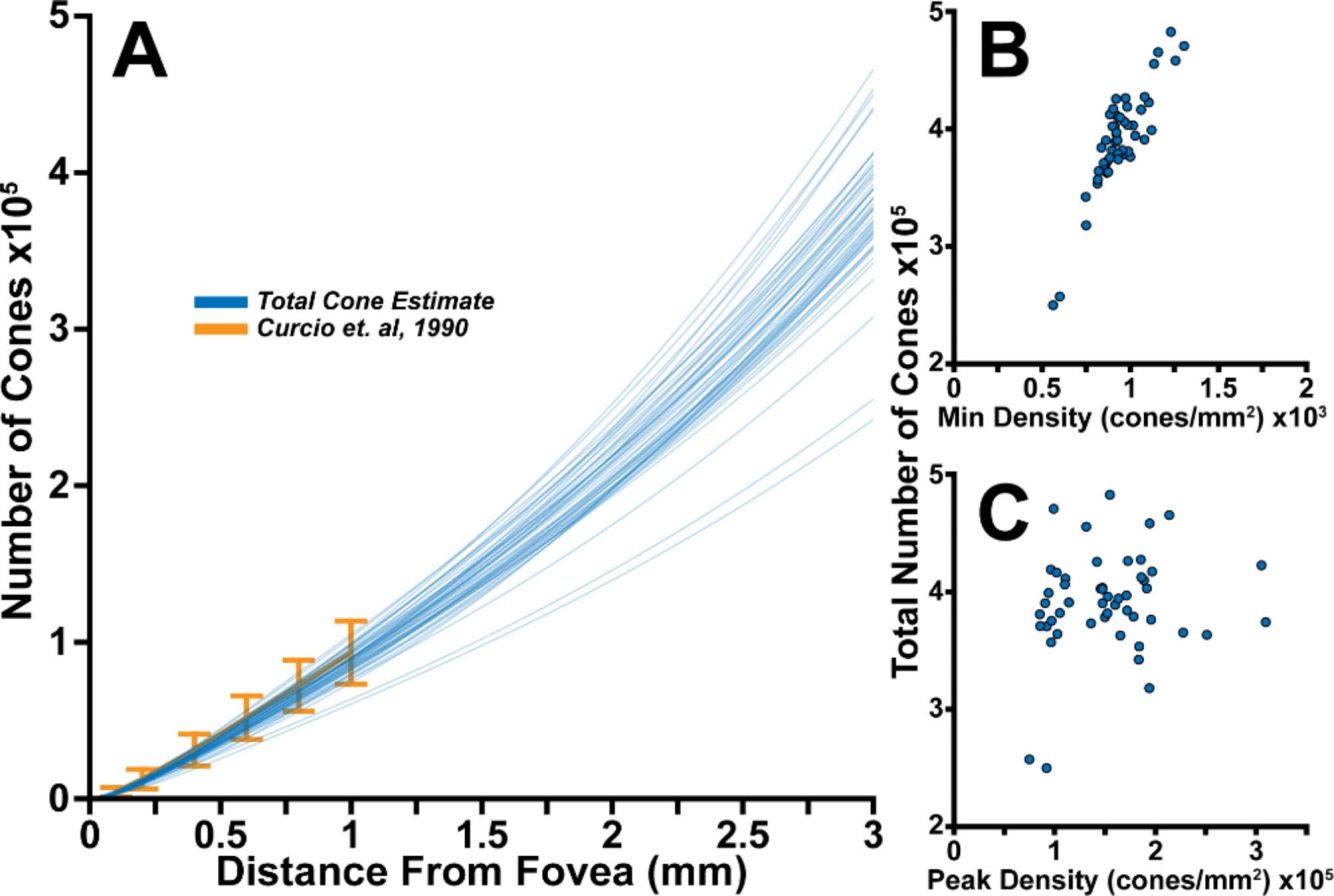
Individual total cone counts as a function of eccentricity (**blue lines**) showed good agreement with histology-based cone counts (**gold line**)^8^, with 96% of our participants’ data falling within the prediction interval (**gold error bars**,** A**). Minimum cone density and peak cone density are plotted versus total cone counts at 3 mm (**B**,** C**). Minimum cone density was correlated with the total number of cones (*r* = 0.89, whereas peak cone density was not.

We extracted peak cone densities (PCD) and minimum densities by fitting each participants’ meridians to a pair of Cauchy distributions with equivalent scales (see “Methods” section). This yielded average (± std dev) PCDs of 152,906 ± 53,209 cones/mm^2^, consistent with prior reports^8,9,13–16^. From these same distributions we estimated the total cones present in each montage. We found that the total number of cones were strongly correlated with minimum density (Pearson *r* = 0.89 CI:0.81–0.93, *p* < 0.001), but not PCD (*p* = 0.24; Fig. 5B**/C**). Minimum cone density was not correlated with PCD (*p*= 0.34). Total cone counts at 1 mm were slightly lower on average than previously reported counts in histology^8^ (93,117 vs. 88,612 cones). However, our population showed significant overlap with their 7 participants, with 96% (48/50) of our participants within the prediction interval of histological data at that retinal location (Fig. 5A).

## Discussion

Here we present a fully automated approach for measuring cone photoreceptor density across AOSLO montages. This approach automatically estimated cone density in all 50 eyes, rarely requiring input from the user 2/50 participants). Our analysis yielded results consistent with prior observations of density and total cone counts, but also allowed us to interrogate more metrics, such as the inter-participant variability and total cones per participants.

Our automated measurements of cone density also enabled estimates of the total cones in our analyzable area. We found that broadly, total cone counts were in agreement with those reported by Curcio et al. over the 0–1 mm eccentricity range^8^. Moreover, prior studies have suggested that while the human fovea features widely varying densities, retina-wide total cone counts are relatively consistent, with most reports (excepting Østerberg’s)^17^featuring total cone estimates around 4 million^8,18,19^. For instance, Curcio et al. observed 3-fold and 1.4-fold ranges at 0.1 and 1 mm respectively in 7 retinas. Our data is consistent with these ranges, with 2.5- and 1.75-fold ranges at 0.1 and 1 mm. While this was consistent with the prior hypotheses of relatively consistent total cone counts across individuals, it was unclear what, if anything, drove the relationship within individuals. In particular, if an individual with higher total cone counts also had a higher PCDs, that would imply that the pressure to form a fovea was roughly equivalent between individuals, and the variability in foveal density was a consequence of the baseline cone count in each individual. Our data did not support this hypothesis, as the PCD was uncorrelated with total cone counts (Fig. 5C). Instead, the minimum cone density was correlated with total cone counts, implying that the increase in cone density concomitant with formation of a fovea is independent of the original cone gradient.

Interestingly, we observed that the standard deviation for cone density was typically greater in the nasal-temporal meridians than the inferior-superior meridians, mirroring average density, and indicating a scale-sensitive relationship between the mean and standard deviation. It is unclear why the horizontal meridians are more variable. This difference cannot be accounted for by our algorithm, as it is not directionally sensitive, nor by the presence of vasculature or other disruptive retinal structures. As our algorithm agnostically assesses cone density in a moving window style, we would have expected greater variability, if any, along the vertical meridians due to the increased presence of vasculature.

Notably, there are a few outliers in our data shown in Fig. 4. These outliers likely occurred from the presence of visible rod photoreceptors or the retinal vasculature. In most of the subjects causing outliers (11101 and 11087, superior outliers, 11076 and 11092 nasal outliers), resolution of the rod photoreceptors resulted in two observable peaks within the pseudopolar log power spectrum. As our algorithm only fits a single peak, this could lead to inaccurate density estimates. Future versions of the algorithm could be modified to detect and properly handle the additional peak caused by the rods. Image quality was considered high for all images, though local regions within an image could have areas of reduced photoreceptor visibility which may also contribute to outlier values.

A major feature of this work is the dataset’s size, coverage, and availability. The scale of this dataset allows us to assess, with an estimate of confidence, what is a ‘normal’ density for any location covered by our merged montage, as well as the expected variance for a given location. The coverage of this dataset (~ 6 mm centered at the fovea) enables direct comparison of data that lie along the meridians, and interpolated estimates of data that lie between them. Moreover, we have made the de-identified data including the montaged AOSLO images and processed montage data (stored as MATLAB .mat files) readily available at https://figshare.com/s/8263d11ac4200b9c3723. We anticipate this will facilitate development of other algorithms, particularly those that require large amounts of training data (as for neural networks) or multiple independent images (as each montage contains ~ 57–111 confocal, split detection, and dark-field image triplets, for a total of 4,039 image triplets).

While these data are consistent with prior reports, there are some assumptions made in their calculation that are important to describe. First, the algorithm itself is based in the Fourier domain, which enables us to extract a direct measurement of cell spacing for each retinal region. To convert this inter-cell spacing value to density, we assume that the cone mosaic is arranged in a roughly triangular lattice. This is a reasonable assumption for individuals without retinal pathology and the algorithm performs well as a result. It is unclear, however, how well the algorithm will perform in the presence of retinal pathology, as loss of photoreceptors could reduce the regularity of the mosaic and potentially break the triangular lattice assumption. Further testing and validation will be needed in such cases and therefore caution should be used if it is applied in montages featuring significant retinal degeneration. Modifications to the algorithm such as a placing a minimum confidence requirement may prove useful for future applications to pathology. In addition, we only included axial lengths to estimate the retinal magnification factor for each individual participant. Further algorithmic development could include additional methods to consider individual optics in determining the retinal magnification^20,21^.

The reference implementation of the automated algorithm discussed in this manuscript was implemented in MATLAB 2023a and is freely available at https://github.com/OCVL/Full-Auto-Density-Mapping under the Apache 2.0 license. Examples of how to run the scripts, as well as instructions on how to reproduce the analyses shown here are also included. Additionally, while the analyses in this work all used linear units (cones/mm^2^, microns), the software is also capable of producing all analyses in terms of angular units (degrees); montage data processed using angular units are provided at https://figshare.com/s/8263d11ac4200b9c3723. As previously reported as best practice, we maintain consistency using either linear or angular units throughout the analysis pipeline^22^. We expect the algorithm will work on images of the photoreceptors acquired with other AO imaging systems, though this idea will need to be tested as more datasets from other groups become publicly available.

## Methods

### Participant recruitment

The Institutional Review Board at the University of Pennsylvania approved the study and the study followed the tenets of the Declaration of Helsinki. 50 participants participated in the observational study following explanation of the procedures and study risks and after giving informed consent. Inclusion criteria included visual acuity of 20/25 or better and no history of ocular disease. Best corrected visual acuity (BCVA) was measured using the Early Treatment Diabetic Retinopathy Study (ETDRS)^23^, axial length was measured using an IOL Master (Carl Zeiss Meditec USA, Dublin, CA). The scale of the retinal images was determined by taking the ratio of the participants’ axial lengths to an average axial length of 24 mm using the method previously described^24^.

## Adaptive optics scanning light ophthalmoscope imaging

The multi-modal adaptive optics scanning light ophthalmoscope (AOSLO) used in this study has been previously described^25,26^. Briefly: An infrared 795 nm super luminescent diode was used to illuminate the retina and three photomultiplier tubes arranged for confocal and simultaneous non-confocal split detection captured infrared retinal reflections^26^. Closed loop adaptive optics (AO) correction was implemented through the use of a wavefront sensor and 97-channel deformable mirror. Participants were cyclopleged with one drop each of Phenylephrine and Tropicamide, a dental impression was used to keep the head fixed relative to the AOSLO imaging system, and participants were instructed to fixate on a fixation target al.igned with the AOSLO imaging system to minimize eye movement. Retinal images were acquired over the central 3.5 × 3.5°surrounding the imaging raster and along all four meridians of the macula out to approximately 10˚. At each retinal location, an image sequence of 150 frames was acquired at a rate of 18 frames per second. The system was focused on the photoreceptor layer, such that the confocal images displayed the waveguided photoreceptor outer segment reflectance mosaic while the split-detection images displayed the photoreceptor inner segment mosaic^1^.

Following image acquisition, AOSLO image sequences were desinusoided using images acquired of a Ronchi ruling with known spacing of 118.1 lines per mm, a reference frame from the image sequence was automatically selected using a custom MATLAB algorithm^27^based on Salmon et al^28^., and 50 frames of the AO image sequence were registered to the reference frame to create a high quality averaged image at each location^29^. Co-localized confocal^30^, non-confocal split detection^26^, and dark-field^31^images from each location were then automatically aligned to each other to form a larger montage using a previously described algorithm^27^. Montages assembled in this fashion were then supplemented with additional registered images as needed to bridge any gaps in the montage. Gaps in the automated montages were most often caused by large horizontal blood vessels in the AO video sequence. Less often, there was a larger than normal jump in fixation. For these cases, lower quality average images (or a single frame of the AOSLO video acquisition) that spanned the gap were used to fill the breaks. Importantly, these images were not retained for further analysis of cone mosaic structure, but instead were used only to ensure the accurate spatial positioning of images relative to the rest of the montage.

## Fully automated analysis of the pointwise density of cone photoreceptors

Once montaged, each registered confocal, and non-confocal split detection image pair was extracted from the montage, maintaining their global placement within the montage. These images were then used as the input to a custom MATLAB algorithm for automatically extracting cone spacing and density over a complete montage. The main features of this algorithm have been previously described^32^. Briefly: We divide an image into a series of overlapping ROIs. Each ROI is transformed using the discrete Fourier transform (DFT) and converted it to a pseudopolar representation of its log power spectrum. We obtained a radial average over a horizontally-oriented 90° wedge and fit the result with a single and dual piecewise first-order exponentials designed to extract the ROI’s modal (cell) spacing. A confidence metric was also derived from this fit based on the residuals of the final fit. A pixelwise weighted average of overlapping ROIs across the montage was calculated to create spacing and confidence maps for the montage. To convert between angular and linear units, we assumed a scale factor of 291 microns/degree for a participant with a 24 mm axial length and then multiplied by a magnification factor for each subject, using each subjects’ axial length in mm divided by 24 mm^33^. Finally, we converted spacing maps to density in millimeters, assuming that on average cells are arranged in a triangular lattice^34^.

In this work, we have enhanced the previously described algorithm to enable peak density finding, eccentricity-dependent ROI sizes, and multi-dataset blending. To find the location of peak cone density (PCD), we first performed the previously described algorithm on montages using a fixed 256 × 256-pixel ROI size. Using the resultant density map, we created a binary mask of all pixel values greater than the 85th percentile. The bounding box of the largest connected component in the binary mask was used to refine the search area. Within the refined region, we smoothed density values with a 2D Gaussian filter (σ = 8), determined the contour at the 85th percentile, and fit the convex hull of the contour with an ellipse. The center of this ellipse was taken as the location of peak density in the montage and used for the remainder of the algorithm as the foveal center. In this way, foveal locations were identified even for cases where the foveal cones were not resolved. As a catch to ensure the proper fovea region is detected, the algorithm checks all contiguous areas of pixels detected with cone densities above the 85th percentile and if any of the smaller areas is more than 40% the size of the largest area the algorithm will prompt the user to draw a box around the fovea and parafoveal region. The algorithm will then proceed as described above using the manually selected region.

While the above was highly effective at finding the location of the PCD, it was often insufficient for obtaining an accurate measure of PCD due to the number of overlapping images at that location. For this reason, we also isolated the confocal image that contained the PCD and analyzed its density separately from the full montages for later integration. The ability to independently analyze and integrate foveal images is provided as an optional step in our reference implementation of the algorithm.

The topography of the normal human retina features high, rapidly varying densities close to the foveal center and lower, gradual changes moving out towards the periphery^8–11^. We used the knowledge of this topography in conjunction with the location of peak density to generate eccentricity-dependent ROI sizes. We used a previously described approach^9^ based on an exponential function to calculate ROI size as a function of distance from the location of peak density. Specifically, we obtained the analytical solution to an exponential function that intersected eccentricities of 1 to 10° at the sizes of 128 × 128 pixels to 384 × 384 pixels, respectively. Eccentricities < 1° were fixed at 128 pixels, and eccentricities > 10° were fixed at 384. This function was used to determine ROI sizes across all images in the montage, resulting in smaller ROIs (finer sampling) close to the region of peak density, and larger ROIs (coarser sampling) further away. In some cases, ROIs extended beyond the valid image area; in these cases, we shrunk the ROI by pixels until it was fully encapsulated by the image.

**Fig. 6:**
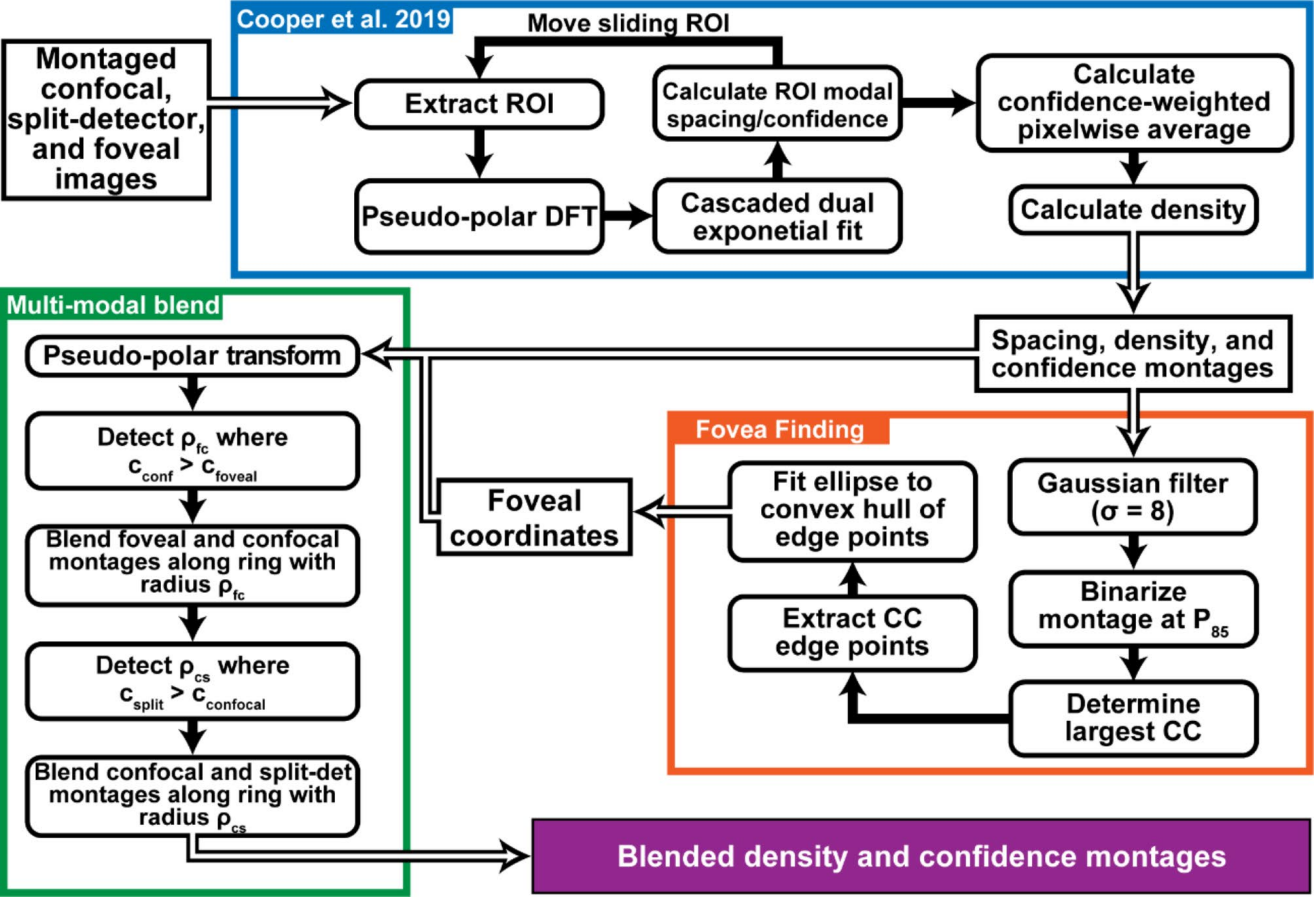
The algorithm used to assess the data. First, the confocal and split-detector montages and foveal images were analyzed using a previously described algorithm (**blue box**). Following that, we estimated the foveal center from the confocal data (**orange box**) and blended to form a single density and confidence montage per participant (**green box**).

To combine data from multiple datasets (the confocal and split-detection modality montages, as well as the high quality foveal image), we first used a stitching algorithm based on the pixelwise confidence from each modality. First, we ran each modality through our algorithm separately, resulting in a pair of density and confidence maps from each dataset. We then rescaled all montages to the smallest micron/pixel scale in the dataset, then calculated the radial average of each confidence map centered on the location of peak density (determined from the dataset with higher confidence at that location). From this, we determined which dataset had higher average confidence as a function of eccentricity. We chose this location as the ‘seam’, or ‘transition radius’ at which one dataset is used over the other. For example, typically our confocal modality had higher confidence close to the location of peak density so its data was used in the radius close to the location of peak density, and vice versa for the split detection modality. To prevent hard seams between the stitched datasets, we linearly blended the values of each dataset over a range determined by when the confidence of either dataset was 0.2 (20%) higher than the other. This process is summarized in Fig. 6.

### Summary of pointwise density and confidence

Before aggregation, each participant’s density maps were filtered to remove low confidence data and outliers. Specifically, we removed regions with confidence values below the montage’s 5th percentile and densities above the median foveal density beyond 400 microns. We flipped all montages as necessary to ensure consistent Nasal/Temporal axes and co-aligned each dataset using their automatically determined locations of peak density. Next, we down-sampled each density and confidence map by a factor of 4 to minimize spatial redundancy and accelerate processing. From these co-aligned, down-sampled maps, we first determined the number of montages that overlapped at each pixel (an “overlap map”), then calculated the average density and confidence at each pixel.

To summarize density and confidence across all participants, we used the overlap map to exclude regions with less than 10 participants. From these regions, we extracted 1° strips along each meridian, and averaged along the short axis of each strip to obtain estimates of density and confidence as a function of eccentricity.

We performed similar analyses on individual participants’ data. We first determined the average density and confidence along each meridian as described above. We then obtained total cone estimates as a function of eccentricity as follows: from each participant’s density map, we extracted a polar average of the horizontal and vertical meridians. Many participants exhibited erroneously low densities close to the fovea as cone spacing at that location approached or surpassed the resolution limit of our device; 20 of the 50 participants had unresolvable foveal cones, and of the remaining 30, 13 were unresolvable with the algorithm. Thus, for 33 of our 50 subjects, we excluded data that was not consistently monotonically decreasing as a function of eccentricity. To interpolate the expected cone densities at this location, we fit a pair of amplitude-linked dual Cauchy distributions to the vertical and horizontal meridians simultaneously. This ensured that both meridians reached the same maximal peak but allowed differing distribution widths to account for slight differences in falloff observed between the vertical and horizontal meridians (Fig. 3A). Moreover, the Cauchy distribution is dual-sided and easily extendable to a bivariate expression encapsulating the falloff differential between the vertical and horizontal meridian, unlike previously used exponential^10^and power fits^35^ which are only analytically defined for univariate data. From these bivariate fits, we estimated the number of cones present in a series of annuli with increasing internal radii. The width of each annulus was fixed at 5 pixels (~ 7 μm); this size was determined empirically to have sufficient size as to provide a reasonable estimate of total cones while not sacrificing resolution. The density in each annulus was divided into horizontal and vertical wedges, and the mean density was determined within the horizontal and vertical wedges. The horizontal and vertical wedge’s mean densities were multiplied by their respective areas and summed to obtain an estimate of the number of cones present in that annulus. A cumulative sum was then calculated over all annuli to obtain a total cone estimate as a function of eccentricity.

## Electronic supplementary material

Below is the link to the electronic supplementary material.

Supplementary Material 1

## Data Availability

The datasets generated during and/or analyzed during the current study are available at the following FigShare location: https://figshare.com/s/8263d11ac4200b9c3723.
